# Evidence for Powassan virus deletions and defective RNA in field-collected ticks

**DOI:** 10.1128/jvi.01356-25

**Published:** 2026-01-21

**Authors:** Samantha J. Courtney, Rose M. Langsjoen, Chasity E. Trammell, Rebecca M. Robich, Heidi K. Goethert, Rebekah J. McMinn, Sam R. Telford, Gregory D. Ebel, Anne Piantadosi

**Affiliations:** 1 Center for Vector-borne Infectious Diseases, Colorado State University 3447, Fort Collins, Colorado, USA; 2 Department of Pathology and Laboratory Medicine, Emory University 189273, Atlanta, Georgia, USA; 3 MaineHealth Institute for Research, Maine Medical Center, Scarborough, Maine, USA; 4 Department of Infectious Disease and Global Health, Tufts University 579988, North Grafton, Massachusetts, USA; 5 Division of Infectious Diseases, Department of Medicine, Emory University 234195, Atlanta, Georgia, USA; University of North Carolina at Chapel Hill, Chapel Hill, North Carolina, USA

**Keywords:** Powassan virus, deer tick virus, tick-borne flavivirus, recombination, deletions, defective virus, defective RNAs, defective viral genomes

## Abstract

Powassan virus (POWV) is a tick-borne flavivirus endemic to the United States, Canada, and parts of Russia. POWV remains an under-studied pathogen, despite the potential for serious and life-threatening neurologic complications following infection. While prior studies have characterized viral diversity due to single nucleotide polymorphisms, little is known about POWV recombination, defective RNAs (D-RNAs), and functional structural variants (SVs). Understanding POWV recombination in its natural vector can provide important insights into its replication and evolution. We analyzed POWV sequence data from 53 ticks collected from the Northeastern United States to characterize and quantify recombination patterns in naturally infected ticks. We then compared these results to single-passage isolates. Deletions were common in POWV RNA from ticks, and several areas of the genome were enriched for recombination junctions. Deletions were often associated with areas of microhomology. While most deletions were sample-specific, two major deletion archetypes were observed across multiple tick samples. The first consisted of small 19–50 base deletions in the methyltransferase domain of the ns5 RNA-dependent RNA-polymerase coding sequence, resulting in a mixture of putative SVs and D-RNAs. The second consisted of approximately 1,600 base deletions spanning the ns2a-ns3 coding sequences, resulting in putative D-RNAs with abrogated viral protease function. Deletions in ns2a-ns3 were significantly enriched after one passage in baby hamster kidney cells, despite a decrease in overall deletions. These results demonstrate that POWV RNA recombines frequently, with certain variants more common than others. These findings may carry implications for virus immune evasion and persistence in ticks.

**IMPORTANCE:**

Powassan virus is a tick-borne flavivirus that can cause serious, life-threatening neurological disease. Understanding how Powassan virus replicates and evolves within its tick vector may elucidate factors important for persistence, transmission, and human disease. Defective RNAs (D-RNAs) are replication-incompetent viral genomes generated through internal deletions. D-RNAs are associated with disease severity and persistent infection in other viruses but have not been described for Powassan virus. Here, we show that Powassan virus produces abundant putative D-RNAs in field-collected ticks and that patterns of D-RNA expression change after one passage in mammalian cells. Although the function of these D-RNAs remains unknown, this work demonstrates that they occur under natural conditions and establishes a critical framework for investigating the role of D-RNAs in Powassan virus replication and transmission.

## INTRODUCTION

Powassan virus (POWV) is a flavivirus within the tick-borne encephalitis virus (TBEV) serogroup that is endemic in the USA, Canada, and the Russian Far East (1–4). POWV is an emerging pathogen of concern (5), given the increase in disease incidence over the past two decades and the propensity of the virus to cause severe and frequently fatal viral encephalitis. While analysis of POWV genome sequences has yielded critical data for understanding POWV genomic epidemiology, spatial population structure, and evolution, few studies have considered the diversity of POWV populations within individual hosts. Still fewer have considered the recombinant components of intrahost populations. Viral RNA recombination can have a profound influence on viral evolution, as well as produce defective virus that can impact viral replication, persistence, and disease severity. Thus, by characterizing viral recombination in relevant POWV hosts, we can determine potential sources of genetic variation and identify novel research avenues for identifying determinants of persistence and virulence.

Recombination is a key driver of RNA virus evolution (6), including mosquito-borne flaviviruses (7, 8). This process involves template switching between distinct RNA strands or within the same strand and can generate deletions and duplications (9) that result in RNA structural variants (SVs) or defective RNAs (D-RNAs; also known as defective viral genomes, DVGs, or defective-interfering RNAs, DI-RNAs) (10, 11). Both SVs and D-RNAs are recombinant viral RNAs with deletions and/or duplications, but they differ functionally: SVs remain autonomously functional, while D-RNAs cannot replicate and/or transmit in the absence of functional complementation by co-infecting intact viral genomes (12–14). Although SVs and D-RNAs were initially thought to be artifacts of *in vitro* passaging, subsequent studies detected and characterized them in animal models of mosquito-borne flaviviruses and alphaviruses (15–17) and natural infection of respiratory viruses (18–20) and flaviviruses (11). D-RNAs in tissue culture can disrupt viral replication and induce host pro-inflammatory responses (21). However, in natural infection, D-RNAs may have variable roles in pathogenesis (21), with some studies showing antiviral effects for SARS-CoV-2 and Zika virus (16, 22), others demonstrating a role in persistent infection for Usutu and Langat viruses (15, 23), and still others suggesting differential roles for transmitted versus *de novo*-produced alphavirus D-RNAs (17). Despite the significant global health impact of tick-borne flaviviruses (TBFVs), our understanding of recombination in these viruses remains limited (24–26), especially the formation of D-RNAs in natural tick vectors. In addition, while recombination has been documented in other flaviviruses (8, 24, 27), few studies have mapped specific regions of microhomology or performed genome-wide deletion analyses, limiting direct comparisons (28).

*Ixodes* ticks transmit POWV via blood meal exchange with mammalian hosts (e.g., shrews, deer mice, groundhogs, squirrels) and humans (1). Relatively little is known about the maintenance of POWV and other TBFVs in mammalian reservoirs, and experimental and modeling studies suggest that TBFVs also can be effectively maintained in tick populations (29, 30). Ticks are unique among arthropod vectors due to their multi-stage, blood meal-dependent life cycle: between each of three active life stages (larva, nymph, and adult), ticks take a single blood meal from a single host before molting into the next stage. POWV has been identified in field-collected ticks of multiple life stages (1, 31) and is maintained through all life stages via transstadial (32–34) and vertical transmission (30, 35), as well as through infected ticks transmitting the virus via an animal’s bloodstream to an uninfected tick via co-feeding (29). Because the tick vector may act as an important reservoir for POWV, it is a critical host in which to study POWV replication and recombination. Accordingly, we sought to expand our knowledge of POWV recombination in ticks naturally infected with POWV lineage II virus, also known as deer tick virus (DTV), to gain insight into viral population dynamics within these critical hosts. We performed detailed recombination analyses to: (i) characterize POWV deletions in naturally infected ticks; (ii) determine potential factors that influence recombination leading to deletions; and (iii) determine whether tissue culture passage affects POWV deletion content.

## RESULTS

### Deletions occur throughout the POWV genome, and most are predicted to result in defective RNAs

We analyzed POWV reads generated by RNA metagenomic short-read sequencing from 53 individual POWV-positive ticks collected from the Northeastern United States between 2018 and 2020 (31) (Table 1; Table S1). Most of the samples (*n* = 33) were collected from Maine in 2018 (*n* = 3), 2019 (*n* = 14), and 2020 (*n* = 16). The remaining samples were collected in either Massachusetts (2018, *n* = 2; 2020, *n* = 6), New York State (2019, *n* = 9), or New Jersey (2019, *n* = 3). All ticks in this study were positive for POWV lineage II, also known as DTV. We refer to this as POWV throughout the manuscript.

**TABLE 1:** Naturally infected ticks included, by year and location

State	2018	2019	2020	Total
ME	3	14	16	33
MA	2	0	6	8
NY	0	9	0	9
NJ	0	3	0	3

**Fig 1:**
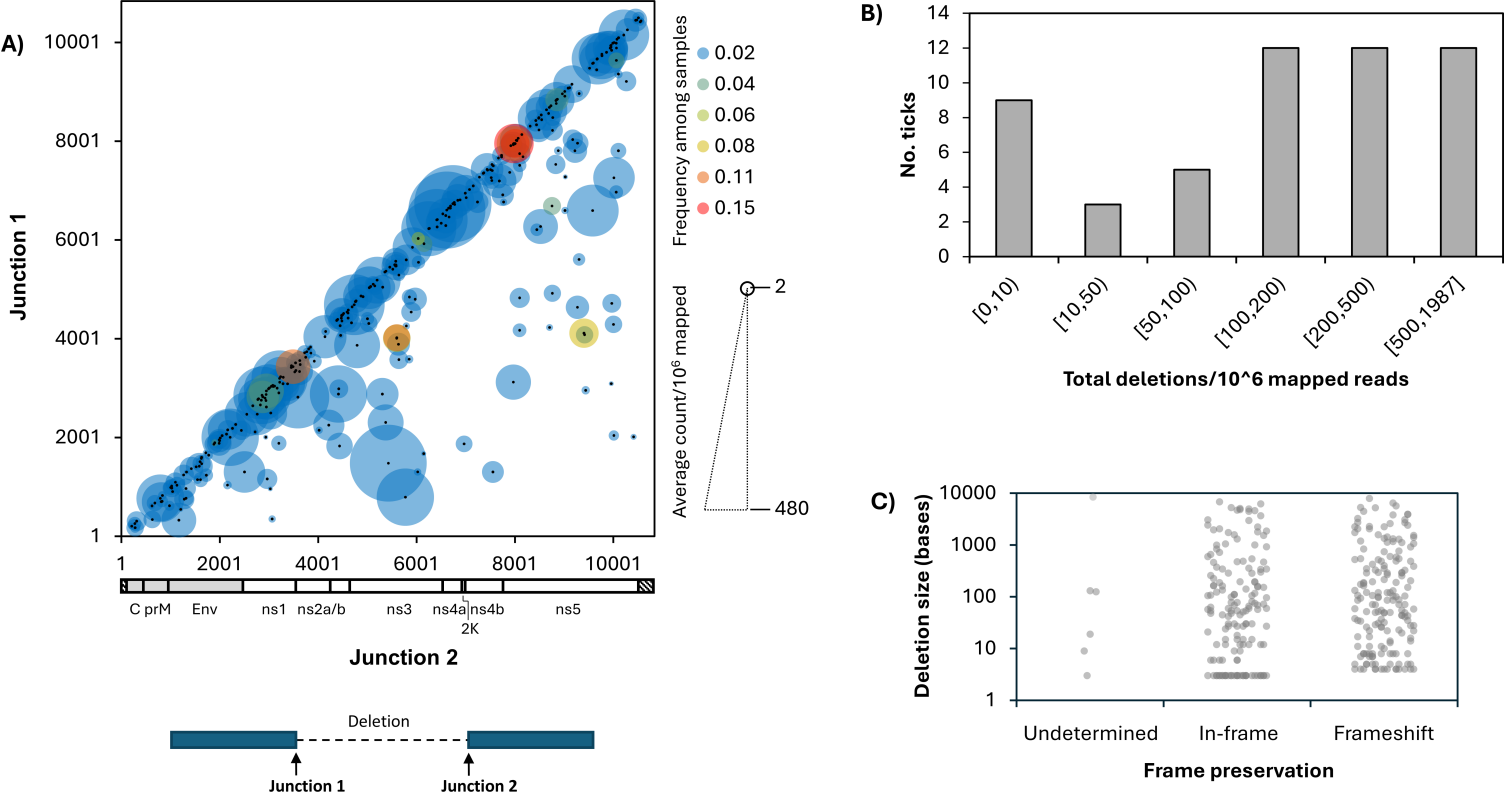
Characteristics of POWV recombination breakpoint junctions and deletions. POWV RNA derived from 53 naturally infected tick samples underwent random-primer cDNA synthesis, Nextera XT tagmentation, and sequencing on an Illumina platform. Recombination events were mapped using Viral Recombination Mapper (ViReMa). Deletions were extracted, and recombination breakpoint junction nucleotide positions (Junction 1, Junction 2) were indexed to the POWV reference HM440559.1. Unique deletions were defined as having a unique combination of J1 and J2 nucleotide positions. (**A**) Nucleotide positions of Junction 1 (y-axis) and Junction 2 (X-axis) for each unique deletion, colored by its frequency among samples and sized by its average read count per million mapped reads within each sample. (**B**) Distribution of the total number of deletions per 10^6^ mapped reads across 53 tick samples. (**C**) Distribution of deletion size (Y-axis) by coding frame (undetermined are deletions occurring between non-coding and coding regions of the genome; in-frame are deletions with a base size divisible by 3, and frameshift are deletions predicted to result in a shift to the coding frame).

Across all samples, we identified 330 unique deletions; that is, deletions with unique nucleotide positions at each recombination junction (Fig. 1). Each deletion was identified within the span of a single sequencing read. We did not rely on contig assemblies to identify deletions. The read lengths for all reads and those containing deletions are presented in Tables S2 and S3, respectively. Most samples contained between 1 and 10 unique deletions (Fig. S1). Most unique deletions were specific to a single sample and ranged in expression from 2 to 480 deletions per 10^6^ mapped reads (Fig. 1A), corresponding to between 0.004% and 35% of the number of reads at a given position (Fig. S1). The observation that most deletions were specific to a single sample suggests *de novo* production within each tick, rather than accumulation over multiple transmission cycles between vector and host, as has been suggested for some mosquito-borne viruses (36). Among all samples, the number of deletions ranged between 0 and 1,986 per 10^6^ mapped reads (between 0 and 0.2% of total reads), with a median of 119 deletions per 10^6^ mapped reads (0.017% of total reads) (Fig. 1B). There was a large number of deletions with a 5′ recombination breakpoint junction (Junction 1, J1) in the envelope (E) coding region, as well as deletions with a 3′ recombination breakpoint junction (Junction 2, J2) in the ns3 coding region (Fig. 1B), suggesting that there could be junction enrichment in specific genomic regions.

Some deletions were observed across multiple samples. The most common included deletions in the methyltransferase (MTase) region of the ns5 RNA-dependent RNA-polymerase (RdRp) coding region, deletions spanning the viral protease (ns2a-ns3), small deletions in ns1, and deletions spanning the majority of the non-structural region between ns2a and ns5 (Fig. 1A). Interestingly, a common deletion archetype previously described for flaviviruses—deletions spanning envelope (E) and ending at the beginning of ns1 (11, 37, 38)—was rare among these samples, with only one such deletion identified in one sample.

Deletions can result in autonomously functional SVs or D-RNAs, also known as DVGs, which require a helper virus to replicate and/or transmit. To evaluate whether the deletions we observed might represent SVs or D-RNAs, we considered their size and coding frame preservation (Fig. 1C). Over half of all deletions found in the POWV single open reading frame (ORF) resulted in a frameshift, suggesting they would result in non-functional proteins and therefore be considered putative D-RNAs (Fig. 1C). A further 23% of all deletions were over 500 nucleotides in length (Fig. 1C) and would be expected to disrupt necessary viral proteins, also resulting in putative D-RNAs. On the other hand, 10% of all deletions observed were only three nucleotides in length, resulting in a single amino acid deletion (Fig. 1C), which would largely be predicted to result in SVs. Small deletions, although unlikely to result from RNA recombination *per se*, are included in subsequent analyses, as they may have a large impact on viral RNA function, including as potential D-RNAs. In addition to deletions, single nucleotide variants may occasionally result in D-RNAs if they result in an early stop codon. As this is a common type of D-RNA for other flaviviruses, such as dengue virus, we analyzed intra-sample single nucleotide variants (iSNVs) in our samples and found four iSNVs that would result in a premature stop codon, out of 334 total iSNVs identified. The position of these did not seem to correspond to peaks in deletion breakpoint junction usage and thus seemed to occur more or less at random (Fig. S2).

### Common deletion archetypes occur in the ns2A-ns3 protease regions and in the methyltransferase domain of the ns5 RNA-dependent RNA-polymerase

**Fig 2:**
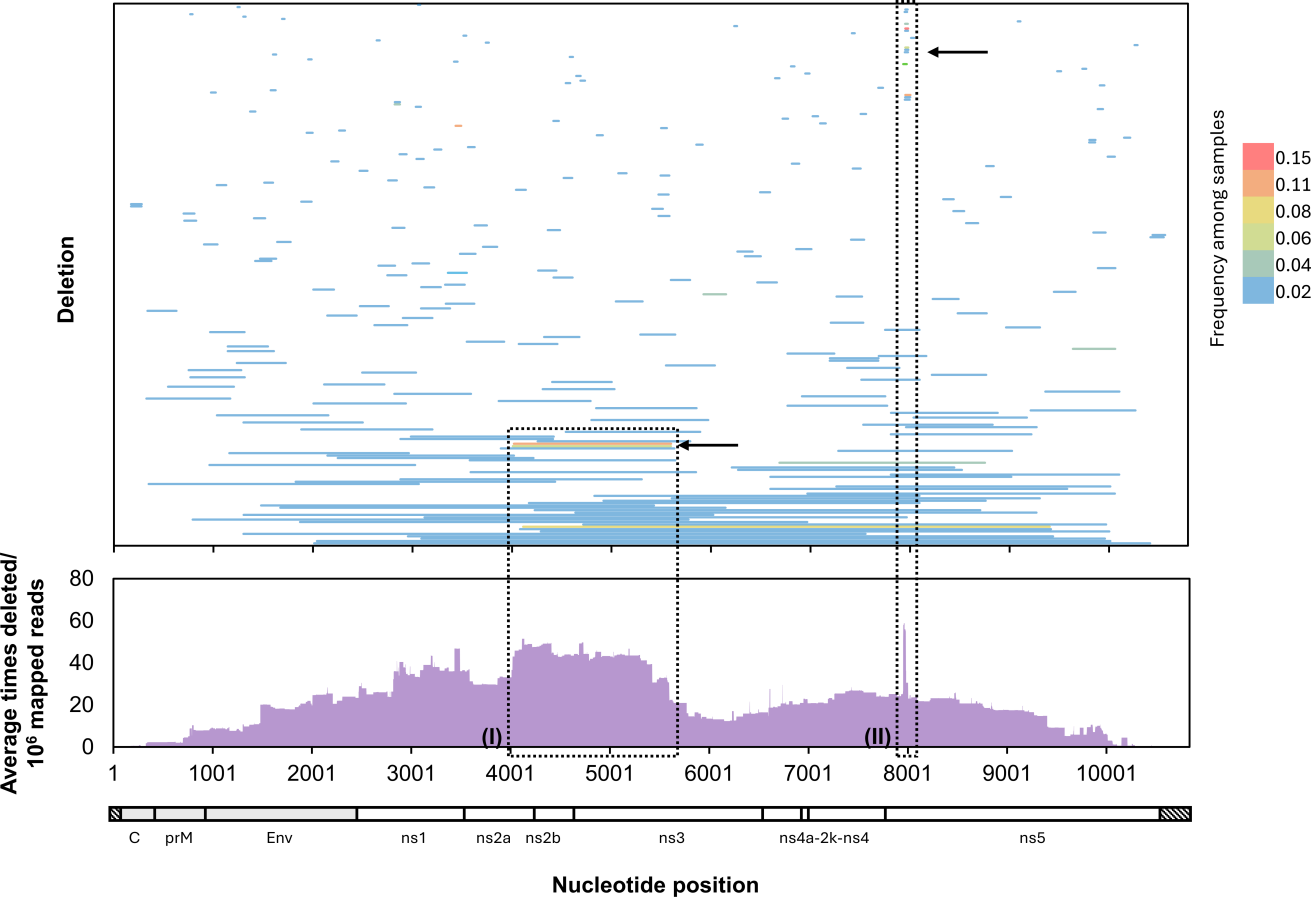
POWV deletion archetypes in naturally infected ticks correspond to peaks in cumulative nucleotide deletion. Individual deletions identified in naturally infected ticks were mapped and colored by frequency across 53 samples (top). For each nucleotide position, the number of times a deletion containing it was found within a read in our data (normalized to total mapped reads) was calculated and averaged across all 53 samples (bottom). Inset (I) highlights a peak in nucleotide deletion frequency across the ns2a-ns3 coding regions that corresponds to two highly represented deletion archetypes with similar boundaries (arrow), and inset (II) highlights a peak in nucleotide deletion frequency driven by small deletions in the methyltransferase region of the ns5 coding region (arrow).

Comparing POWV deletion positions between samples, we identified specific recombination patterns that were present in multiple ticks (Fig. 2). One common archetype shared similar breakpoint junctions in the ns2A gene between nucleotides 3889 and 4259 (J1) and in the ns3 gene between nucleotides 5597 and 5793 (J2). Two specific deletions occurred frequently among samples with this archetype: one with J1^J2 junctions at 4020^5599 and the other with J1^J2 junctions at 4010^5597 (Fig. 2 top**,** inset I). These two deletions were collectively present in almost 20% of all tick samples at estimated frequencies between 0.07% and 3.4% (between 2.3 and 204.5 count/10^6^ mapped reads) and also corresponded to a region of the genome that had an overall higher number of deletions detected within our short-read data (Fig. 2 bottom**,** inset I). All deletions of this archetype (J1 in ns2a and J2 in ns3) are expected to result in the partial removal of the ns3 viral protease as well as the complete removal of its viral cofactor ns2B and are therefore predicted to function as D-RNAs. However, the potential functions of the putative D-RNAs identified in this study and their potential impact on viral replication have not been assessed.

The most common single deletion, identified in 15% of all samples, had J1^J2 nucleotide junctions at 7953^7981 in the methyltransferase (MTase) region of the ns5 coding region (Fig. 2 top**,** inset II), resulted in a 9-amino acid in-frame deletion, and occurred at frequencies between 0.2% and 8% (between 13.1 and 604.5 count/10^6^ mapped reads). In addition, we detected 11 other deletions with similar junctions, all with J1 between nucleotides 7932 and 7954 and J2 between nucleotides 7968 and 8004. Because they all occur in the same area of the MTase coding region, we refer to this deletion archetype as “MTase deletions.” At least one MTase deletion between 19 and 50 bases in length was identified in over 30% of samples (Fig. 2 top). These MTase deletions corresponded to a peak in overall deletion frequency across all samples (Fig. 2 bottom**,** inset II). Interestingly, while five MTase deletions are predicted to result in an SV with an in-frame nine amino acid deletion, including the most common MTase deletion with J1^J2 nucleotide junctions at 7953^7981, seven others result in 22–49 nucleotide frameshifts and are therefore predicted to result in putative D-RNAs. In-frame and frameshift deletions occurred at similar frequencies, between 0.04–8% and 0.03–7%, respectively. The deletions occur in a region of the MTase domain that is believed to interact with the RNA-dependent polymerase (RDP) domain in a predicted model of the POWV RdRp (Fig. S3). Deleting amino acids at the MTase-RDP interface in Japanese encephalitis virus (JEV) results in significantly decreased, though intact, replication (39), so it is possible that in-frame deletions here would have a similar impact.

### POWV deletions occur at sites of microhomologies between nucleotide junctions

**Fig 3:**
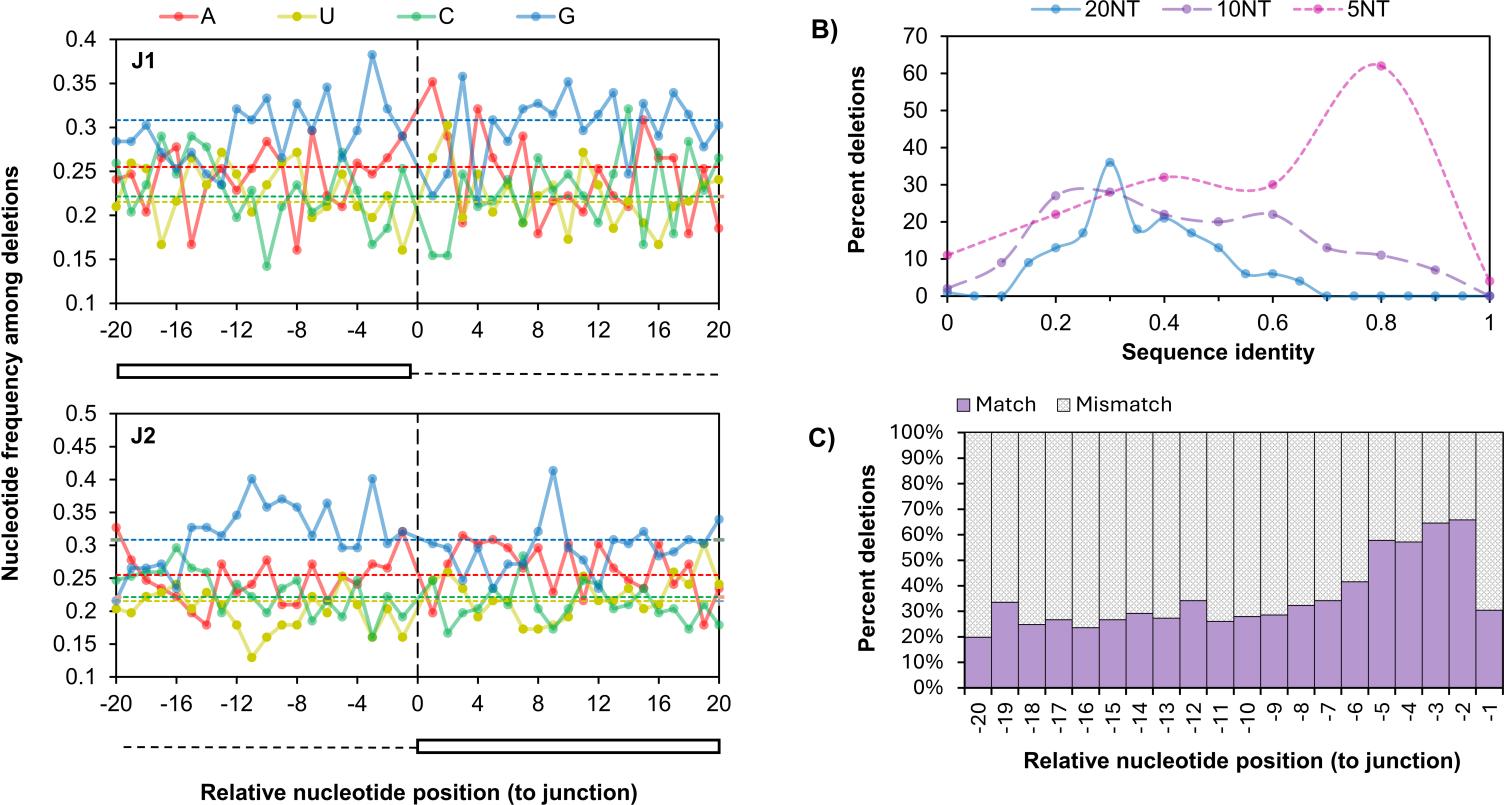
Sequence characteristics at recombination breakpoint junctions identified in naturally infected POWV ticks. (**A**) The distribution of bases (solid lines) within 20 bases up- and -downstream of recombination Junctions 1 (J1; top) and 2 (J2; bottom) compared to the relative distribution of each base across the POWV genome (dashed lines). (**B**) Percent of deletions (Y-axis) with nucleotide sequence identities (X-axis) between Junction 1 (the sequence observed upstream of J1 present in the read) and J2 (observed upstream of Junction 2 present in the reference) within 5 (pink), 10 (purple), and 20 (blue) nucleotides of the recombination junction. (**C**) Percent of deletions with a match (purple) or mismatch (gray) at each nucleotide position relative to the recombination junction.

To assess the role of nucleotide sequence in POWV recombination, we evaluated the nucleotide composition and sequence identity at recombination junctions for deletions ≥100 bases in length (Fig. 3). Smaller deletions were excluded in order to avoid biases from differential mechanisms regulating small, local deletions, such as polymerase slippage. While a slight enrichment of Us was observed immediately downstream of J1 (Fig. 3A, top) and a slight enrichment of Gs was observed upstream of both junctions (Fig. 3A, bottom), no specific nucleotide sequence or motif was associated with either junction. However, we found evidence for high nucleotide sequence identity between the five nucleotides upstream of J1 and the five nucleotides upstream of J2 in most deletions (Fig. 3B and C). This suggests that microhomologies between breakpoint junctions play a role in template selection during POWV RNA recombination for many, but not all, deletions. This is consistent with previous studies, which found that dengue virus (DENV) deletions were frequently associated with small microhomologies (40).

### POWV deletions in ns2A-ns3 are enriched after one passage in tissue culture

**Fig 4:**
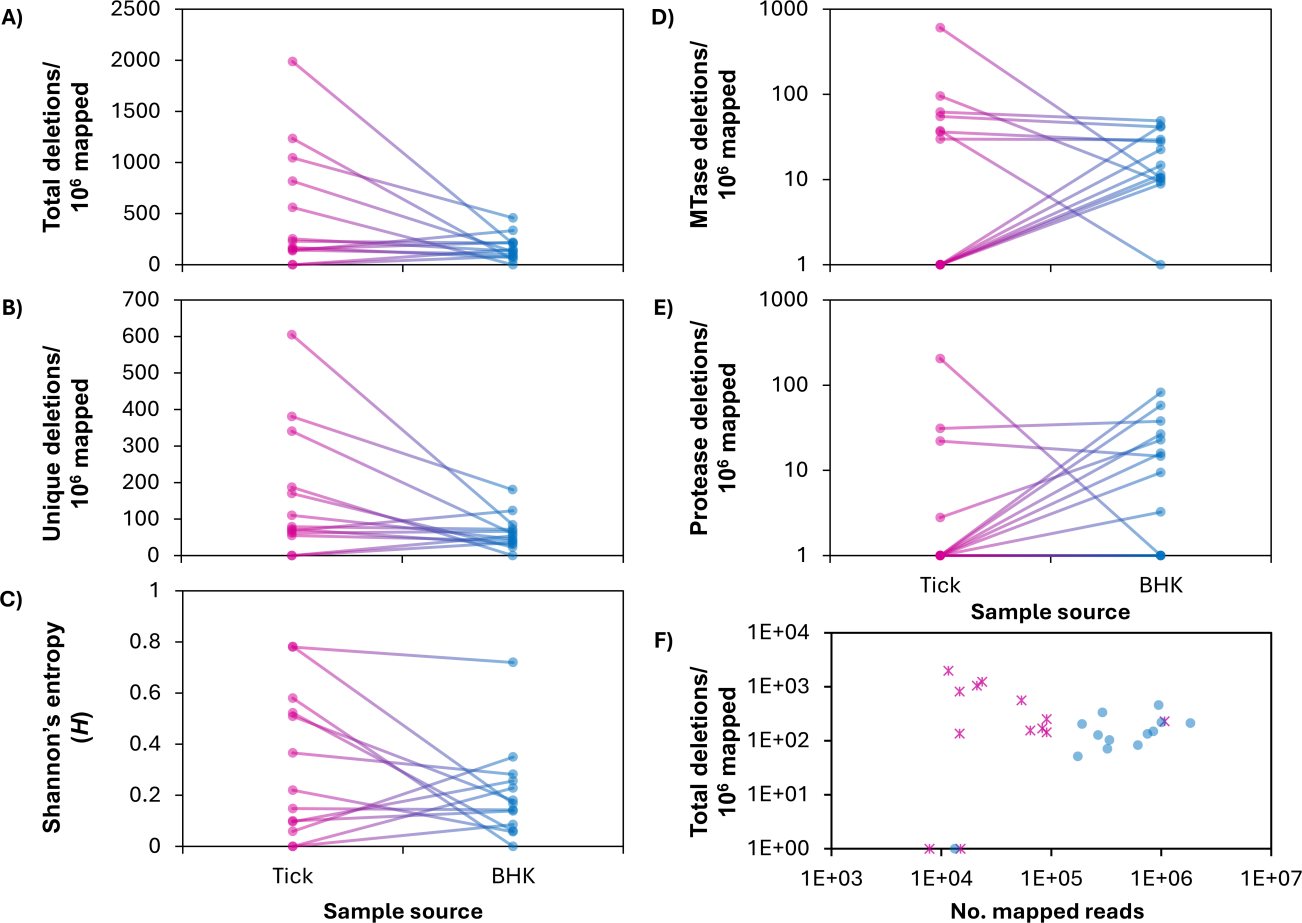
Comparison of POWV deletions between naturally infected ticks and single passage tissue culture supernatants. POWV RNA from 13 naturally infected ticks (tick) and RNA derived from the same virus after one passage in baby hamster kidney cells (BHK) was sequenced, and deletion content was determined by recombination mapping. (**A**) Total number of deletions per million mapped reads. (**B**) Number of unique deletion species per million mapped reads. (**C**) Diversity of deletion species, as determined by Shannon’s entropy (*H*). (**D**) Total number of MTase deletions (small deletions between 19 and 50 bases in length between nucleotide positions 7932 and 8004) per million mapped reads. (**E**) Total number of ns2a-ns3 deletions (approximately 1,600 base deletions occurring between the ns2a and ns3 genes) per million mapped reads. The total number of deletions per million mapped reads was also compared to the total number of reads per sample (**F**), and the two were not significantly correlated (Kendall test, tau = 0.006, *P* = 0.96)

Because tissue culture passaging has been reported to increase D-RNAs in other viruses (31), we compared deletions between viruses from 13 naturally infected ticks and their corresponding culture supernatants after one passage in baby hamster kidney (BHK) cells. We did not observe a statistically significant difference in the number of unique deletions (Wilcoxon rank test, *P* = 0.064; Fig. 4A) or the total number of deletions (Wilcoxon rank test, *P* = 0.11; Fig. 4B), though both tended to decrease after a single passage. Although there was no difference in expression of MTase deletions (Wilcoxon rank test, *P* = 0.6; Fig. 4D), there was a trend toward higher expression of deletions in ns2A-ns3 after passage (Paired-Sample Sign Test, *P* = 0.077, Fig. 4E). Sequencing depth was not correlated with normalized deletion counts (Kendall test, tau = 0.006, *P* = 0.96; Fig. 4F), so we do not expect this to be a confounding variable.

**Fig 5:**
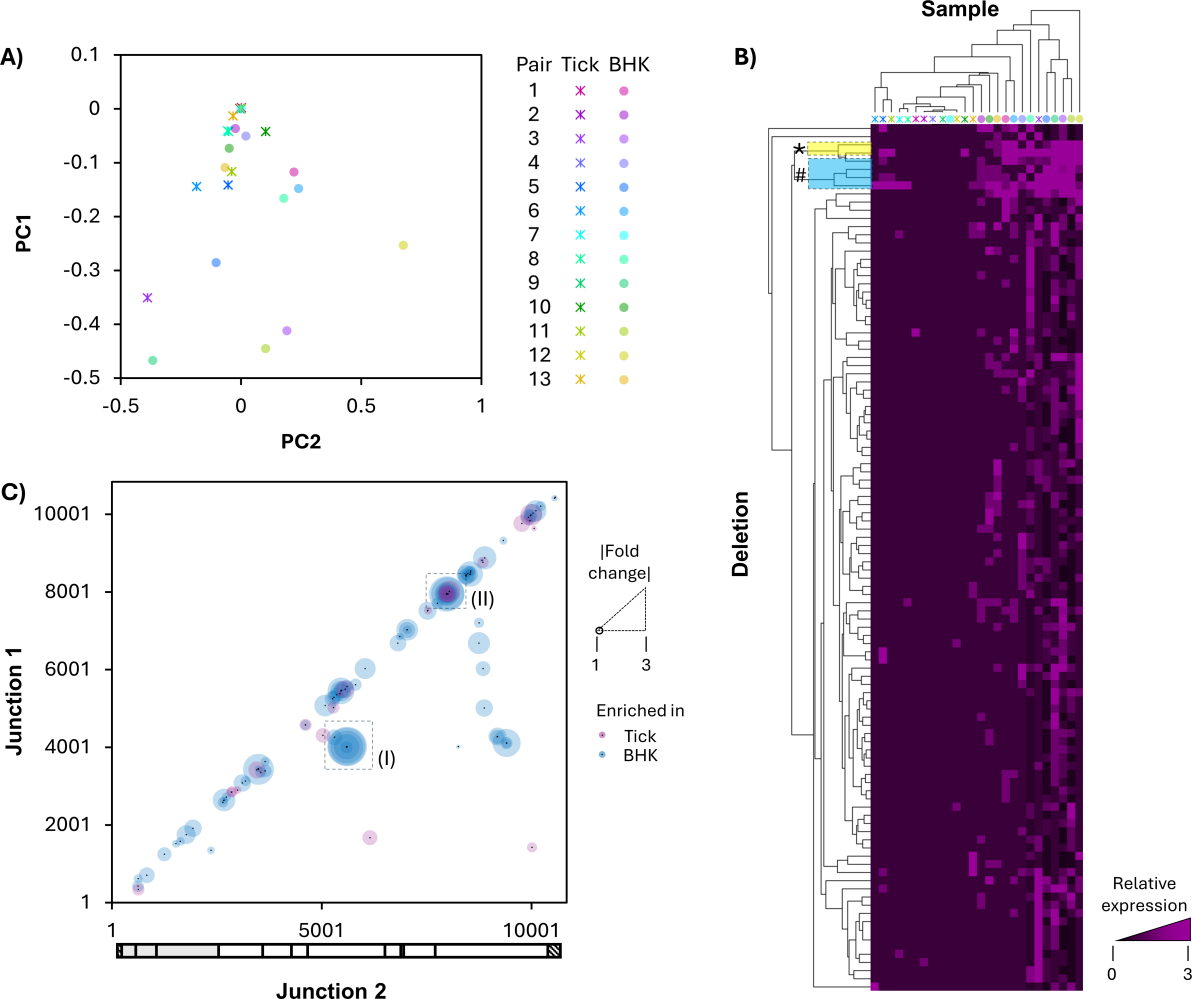
Differential expression of POWV deletions between 13 naturally infected ticks and the same virus after one passage in tissue culture. Differential gene expression of individual deletion species was evaluated using DESeq2 software. (**A**) Principal component analysis (PCA) was performed, and eigenvalues for components 1 (Y-axis) and 2 (X-axis) were plotted for both tick (stars) and BHK (circles) samples and colored by tick/BHK sample pair. (**B**) Hierarchical clustering was performed with clustering by sample (X-axis) and deletion (Y-axis). (**C**) Each deletion species included in DESeq2 analysis was plotted by recombination junction (Junction 1, Y-axis; Junction 2, X-axis), colored by enrichment by compartment (pink, tick; blue, BHK), and sized by log2 fold change. Inset (I) highlights ns2a-ns3 deletions, while inset (II) highlights MTase deletions.

To determine if specific deletions were differentially expressed between naturally infected ticks and single-passage isolates, differential gene (deletion) expression analysis was performed in DESeq2 (Fig. 5). Principal component analysis (PCA) revealed that 12/13 tick samples clustered closely together based on deletion expression, while 5/13 BHK samples clustered closely with tick samples, and the remaining 8/13 BHK samples showed little to no clustering pattern (Fig. 5A). Additionally, very few tick/BHK sample pairs clustered closely with one another. This suggests that, when excluding deletions specific to a single sample, tick samples are more similar to each other than to BHK samples. Hierarchical clustering analysis supported this result, with two naturally infected tick sample groups clustering closely together and multiple BHK groups clustering more distantly (Fig. 5B). Finally, when evaluating differential expression of specific deletions, two deletions had higher expression in BHK cells than tick samples: an in-frame 1,578 base deletion between nucleotides 4020 and 5599 (fold change = −2.8, padj = 0.03; Fig. 5B inset *, Fig. 5C inset I) and a 1,582 base frameshift deletion between nucleotides 4020 and 5603 (fold change = −3, padj = 0.04; Fig. 5B inset *, Fig. 5C inset I). Both are deletions in ns2A-ns3 and are predicted to function as D-RNAs. Three other deletions—two MTase deletions (Fig. 5B inset #, Fig. 5C inset II) and one deletion in ns2A-ns3 (Fig. 5B inset #, Fig. 5C inset I)—were more highly expressed in BHK cells, although this difference was not statistically significant (Fig. 5C). No specific deletions were significantly more highly expressed in tick samples than BHK samples.

### ClickSeq confirms ns2A-ns3 deletions in POWV from tick samples

**Fig 6:**
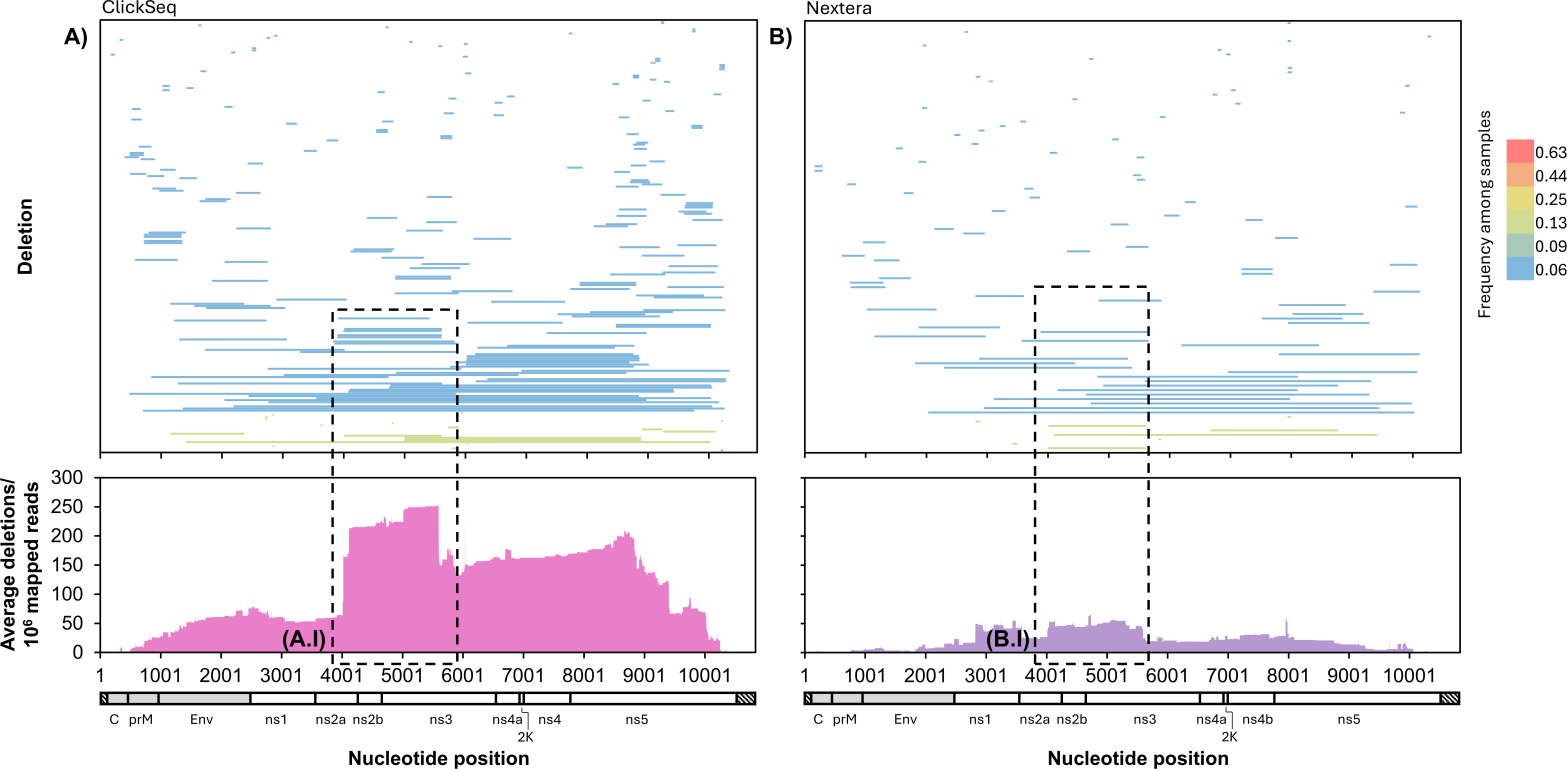
Comparison of POWV deletion expression using two different library preparation methods. POWV RNA from 16 naturally infected tick samples was sequenced using ClickSeq (**A**) and a metagenomic approach (**B**). Individual deletion species were mapped by genome position and colored by frequency across samples (top). For each nucleotide position, the number of times it was deleted in each sample was calculated and averaged across all 16 samples (bottom). Insets A.1. and B.1. indicate deletions in ns2a-ns3, with a Junction 1 breakpoint position between nucleotides 3889 and 4259 and Junction 2 breakpoint position between nucleotides 5597 and 5793.

The above analyses used data from RNA metagenomic sequencing libraries, which were generated through random primer cDNA synthesis and Nextera XT tagmentation (Illumina). To assess the reliability of this technique for recombination detection, we made additional libraries from 16 of the samples (those with available RNA remaining) using ClickSeq, a library preparation method specifically designed to reduce artifactual recombination events (41, 42). Analysis of ClickSeq libraries confirmed the presence of ns2a-ns3 deletions with J1 between nucleotides 3889 and 4259 and J2 between nucleotides 5597 and 5793, including the 4020^5599 deletion, the 4010^5597 deletion, and one other previously identified deletion, as well as five additional unique deletions in this region, which occurred at frequencies between 0.02% and 2% (Fig. 6A and B; insets A.I and B.I). We were not able to confirm the presence of MTase deletions because RNA was not available from the samples with high levels of MTase deletion expression; however, 4 of the 16 samples that were analyzed using ClickSeq had MTase deletions at low levels (Fig. 6).

## DISCUSSION

Deletions are common in RNA viruses and are thought to result from copy-choice, non-homologous RNA recombination; they can result in D-RNAs, dysfunctional RNAs that can affect wild-type virus replication, as well as autonomously functional SVs. Despite the importance of characterizing deletions in RNA viruses, little work has been done to characterize deletions and intrahost recombinant RNAs of TBFVs, especially in the natural tick vector. Here, we found that POWV, a TBFV, produces frequent deletions with a range of sizes throughout the genome, leading to both putative D-RNAs and SVs. We found evidence for small 2–5 base homologies between recombination junctions, suggesting that microhomologies may impact template selection during recombination. Importantly, we found several deletion archetypes that were present in multiple samples, including deletions in the ns2a-ns3 regions spanning the viral protease and its cofactor ns2B, as well as small deletions in the methyltransferase (MTase) region of the ns5 coding region. The deletion expression did not increase after a single passage in tissue culture and rather tended to decrease, except for ns2a-ns3 deletions, which tended to increase after a single tissue culture passage. Finally, we validated the presence of deletions using a recombination-specific sequencing method. In all, our results demonstrate that POWV produces deletions consistent with SVs and D-RNAs in its natural tick vector, and that some deletions arise repeatedly across multiple samples.

While RNA virus recombination and deletions have been described in mammalian hosts, little work has been done to characterize recombinant populations in arthropods. Still, fewer studies have considered recombinant RNAs in tick hosts. Phylogenetic studies of TBEV and louping ill virus (LIV), both close relatives of POWV, have indicated potential recombination events in the evolutionary history of these viruses (24–26, 43, 44), although it is not known in which host they may have occurred. Mosquito-borne flaviviruses have shown different proclivities for RNA recombination and deletion, both *in vitro* and *in vivo*. D-RNAs consisting of deletions between the prM and ns1 genes have been described for West Nile virus (WNV) in lorikeets (11), JEV (38), and Murray Valley fever virus (37) in tissue culture, where they have been associated with inhibition of wild-type virus replication (11) and establishment of persistent infections (37, 38). Although the function of the POWV deletions identified in this study remains unclear, our identification of deletions in POWV from naturally infected ticks recommends further exploration of the role of recombination in TBFV evolution and pathogenesis.

We also show that POWV deletion archetypes arise independently in different tick samples. Prominent among these were deletions spanning the ns2a and ns3 regions, which would disrupt the viral protease and its cofactors and are predicted to function as D-RNAs. Our results also suggest that expression of deletions in ns2a-ns3 increases after one passage in mammalian cells. It is thus interesting to consider whether these deletions may play a host-specific role during POWV replication or are simply by-products of processes critical to replication, such as RNA-RNA and/or protein-RNA interactions. For example, previous studies have shown that the TBFV protease triggers cellular antiviral responses through its interaction with human TRIM-5α for TBEV and Langat virus (LGTV), but not for POWV (45). While specific TRIM-family proteins and their functions are highly host- and virus-specific (46), it is possible that deletions in this region may help POWV in evading mammalian TRIM-mediated immunity. This recommends the comparison of D-RNA populations between TBEV, LGTV, and POWV to determine potential roles for protease deletions during viral replication.

Our provisional results suggest that isolating POWV on BHK cells (i.e., a single passage from ticks to these cells) resulted in lower overall deletion content in isolated virus compared to virus in ticks. The current paradigm is that D-RNAs accumulate over high-multiplicity of infection (MOI) tissue culture passage. It is possible that a low MOI enriched the wild-type population in this case, but the MOI is unknown, as whole tick homogenate was used for isolation in the absence of quantitative assessment of infectious titer. There are also several potential hypotheses for this result. The first is that tick homogenates include both intracellular and extracellular viruses, and the decrease in deletions in cell culture is driven by a lack of non-packaged virus in supernatant. Previous work with influenza virus (47) and multiple alphavirus species (17) has shown that intracellular viral populations are more recombination-rich than extracellular populations in low-passage tissue culture experiments, as well as in a mouse model of chikungunya virus infection (17). Recombinant POWV populations may likewise be enriched intracellularly. This hypothesis can be explicitly tested experimentally by comparing intracellular and extracellular recombinant POWV RNA populations in similarly designed passaging studies or in carefully designed *in vivo* models of infection.

The second potential hypothesis for the observed overall enrichment of deletion expression in ticks, although less supported by current studies, is that POWV D-RNA expression may be associated with persistent replication in the tick vector. Ticks only take blood meals preceding transitions from larval, nymphal, and adult life stages, necessitating persistent infection by TBFVs across life stages for effective transmission to a new host. TBEV has been shown to establish persistent infection in both ticks (32, 48) and tick cells (48), with little evidence of significant genetic variation at the consensus level between the parental virus and the virus after up to 120 days persistence in *Ixodes* ticks. While previous work has not evaluated the relationship between D-RNA expression and persistence in tick hosts, persistence of LGTV in human embryonic kidney cell culture has been positively associated with the accumulation of defective particles (23). Thus, D-RNA expression among POWV populations in ticks may be associated with persistent infection of the tick vector, either as a regulator of persistent infection or a byproduct thereof. Although speculative, this hypothesis could be expressly tested by characterizing D-RNA expression over the course of persistent POWV infection in laboratory-infected ticks.

The primary limitation of our study is the use of POWV sequencing data from prior studies not explicitly designed to detect recombination events. We therefore used ClickSeq, a library preparation method designed for accurate and specific detection of recombination, to validate our findings for a subset of samples with residual RNA available. To ensure a rigorous analysis, we only used data from samples that had a minimum average sequencing depth of 100×. Above this threshold, we did not observe a relationship between higher sequencing depth and an improved ability to detect recombination. Further, deletions with only one read were removed in order to remove potential artifacts generated through either the library preparation process or the Illumina platform. An additional limitation is that our analyses were performed with short-read sequencing data. Further work is needed to evaluate whether the deletions we detected are present in full-length virus genomes, whether multiple deletions co-occur on the same RNA molecule, and whether they may be packaged and released as defective particles. Finally, the role and origin of small deletions, such as single amino acid deletions, may differ from other deletions, and ultimately, functional studies would be required to confirm their role in the viral population. These small deletions were included here for their potential impact on RNA function, particularly frameshift deletions that would result in defective transcripts, but small in-frame deletions may represent typical variance in the population.

In all, the data presented here offer foundational insight into recombination and deletion within POWV, with possible relevance to other TBFVs. Future studies are needed to understand the potential role of deletions and putative D-RNAs during infection and transmission in both *in vitro* and *in vivo* models of ticks and mammalian hosts. Through continued study of POWV recombination and deletion generation, we can gain further insights into this important pathogen’s evolution and pathogenesis.

## MATERIALS AND METHODS

### Samples and metagenomic sequencing

RNA from POWV-positive tick homogenates and single-passage isolates underwent full POWV genome sequencing as part of previous studies (31, 49, 50). Briefly, *Ixodes scapularis* ticks were collected between 2018 and 2020 from multiple survey sites in the Northeastern United States. Whole ticks were bead homogenized and screened for POWV as previously described (31). For some samples, individual tick homogenate was placed onto a monolayer of BHK cells, and supernatant was harvested at 50% cytopathic effect. RNA extracted from tick homogenates and culture supernatants was sequenced using random primer cDNA synthesis followed by Nextera XT (Illumina) library preparation and Illumina sequencing. Recombination analysis was limited to data from samples with a minimum average sequencing depth of 100× across the POWV genome.

### ClickSeq library preparation

Samples for ClickSeq were selected based on availability of residual total nucleic acid (TNA). TNA from tick homogenates was treated with DNase (ArcticZymes) according to the manufacturer’s instructions, and ClickSeq libraries were generated as previously described (17, 42). Briefly, RNA was primed with 1 µL of a 100 µM primer consisting of a random hexamer and partial Illumina p7 adapter sequence, plus 1 µL of a 1:35 azidoNTP:dNTP mix (ClickSeq Technologies), followed by first-strand synthesis with Superscript III enzyme (Invitrogen). Template RNA was removed using RNase H (Invitrogen), first-strand cDNA was bead-purified (Ampure XP, Beckman Coulter), and the full p5 sequencing adapter with a 12-nucleotide unique molecular identifier was added to the first-strand cDNA via Click reaction. The click reaction proceeded at room temperature for 2 h, with 2 µL of a 5 µM p5 oligo, “ClickMix” (ClickSeq Technologies), and ClickCatalyst (ClickSeq Technologies) prepped with 10 mM vitamin C (Biotechne). “Clicked” cDNA was PCR amplified for a total of 21–24 cycles using OneTaq (New England Biolabs), a universal p5 primer, and an i7 indexing primer. Final libraries were run on a 2% agarose E-gel (Invitrogen) and gel-extracted from the 250–650 bp range using the Zymo Gel DNA Recovery kit (Zymo Research). Libraries were quantified by Quant-iT PicoGreen assay (Invitrogen), pooled, and sequenced on a NextSeq 2000 (Illumina).

### Bioinformatics and recombination analysis

Adapters were removed from reads by Illumina’s BaseSpace software. Raw FASTQ files were processed using fastp version 0.23.2 (51) to: (i) ensure complete adapter removal; (ii) quality filter, deduplicate, and filter reads less than 50 bases in length; and (iii) merge overlapping reads and write non-overlapping reads to separate read1 and read2 files. For ClickSeq libraries, fastp was additionally utilized to trim and append UMI sequences to read names. Because paired-end sequencing was performed and recombination mapping tools were designed for single-end data, read names in overlapping read, non-overlapping read 1, and non-overlapping read 2 files were tagged with “/3,” “/1,” and “/2,” respectively, and all read files were concatenated into one FASTQ file for analysis. Reads from tick samples were first mapped to the *Ixodes scapularis* genome using hisat2 v 2.2.1 (52) to remove tick reads. Then, for each sample, a sample-specific POWV consensus sequence was generated by mapping POWV reads to reference sequence HM440559.1 and using pilon (53) to generate a reference-based consensus sequence. POWV reads from each sample were aligned to the sample-specific consensus sequence, and recombination events were identified using ViReMa version 0.25 (54). Deletions were extracted from resulting BED files, and recombination breakpoint junctions were indexed to the HM440559.1 genome. To assess whether deletions were caused by library preparation artifacts during the random primer cDNA synthesis and Nextera XT approach, the length of each read split was calculated (where split 1 is the length of the 5′ end to the recombination junction, and split 2 is the length of the recombination junction to the 3′ end). The distribution of lengths for split 1 was compared to the distribution of lengths for split 2, and these distributions were found to be similar (Fig. S4B), indicating that deletions occurred throughout the length of the read. Similarly, to assess for potential artifacts causing deletions in ClickSeq reads, the lengths of each read split (5′ end to recombination junction and recombination junction to 3′ end) were compared and found to be similar (Fig. S5). Single nucleotide variants were called by mapping reads to the pilon-generated consensus from the sample using bowtie2 with default local settings, followed by variant calling using LoFreq 2.1.5. Additionally, the relative read position of each variant was determined using a pileup generated by bam-readcount, and variants with a relative read position less than 0.4 were removed from the final data set. Variants were annotated using a custom annotator specific to each respective viral genome.

### Protein modeling

The amino acid sequence of the POWV strain NFS001 (Genbank HM440559.1) non-structural protein five was submitted to Expasy/SWISS-MODEL (55) to identify closely matching amino acid sequences available in the Protein Data Bank (PDB) using the HHblits tool (56). The structure for the JEV ns5 was identified as a template (4K6M.1.A) (39), with 57% sequence identity and 99% sequence coverage. A model was subsequently built in ProMod3 v3.2.1 (57) via SWISS-MODEL with a resulting QMGE score of 0.84 and a QMEANDisCo of 0.80 ± 0.05. The molecular surface for the resulting model was calculated in Swiss PDB Viewer v4.1 (58), and amino acids were colored by associated nucleotide deletion data.

### Statistics

For analyses comparing total and unique deletions counts, deletions with only one read count were removed, and raw counts were normalized to count/10^6^ mapped reads. All statistical analyses were performed in RStudio version 2023.6.2.561 (59) using R version 4.3.1 (60). Normality was assessed using Q-Q plots in ggpubr version 0.6.0 (61). The relationship between total read count and either Shannon’s diversity index (H) or total deletion expression was assessed by Kendall correlation analysis. Analysis of variance for multiple groups was performed using the Kruskal-Wallis test, and paired sample analysis was performed using the Wilcoxon rank-sum test. For data that did not fit one or more assumptions of the Wilcoxon rank-sum test, a two-sided paired sample sign test was employed using the EnvStats package for R with mu = 0 and a confidence interval of 95%. Differential gene expression was assessed using DESeq2 v1.40.2 (62); for this, all deletions were pooled across samples, and only deletions with at least two counts in at least two samples were included. Raw read counts were used for analysis, without normalization to total mapped reads, as DESeq employs an internal normalization method. The program was run using paired data structure and a local regression fit. DESeq2 output was used to perform principal component analysis using the prcomp() function and for hierarchical clustering analysis in Cluster 3.0 (63). Clustering using average linkage is shown here, although all linkage algorithms resulted in highly similar results. Hierarchical clustering results were visualized in TreeView (64).

## Data Availability

All raw sequencing data are available in the Sequence Read Archive (SRA) under BioProject PRJNA1011342; all accession numbers available in Table S1.1.

## SUPPLEMENTAL MATERIAL

Fig. S1 jvi.01356-25-s0001.tif Distributions of the number of unique POW deletions across ticks and the frequency of each unique deletion.

Fig. S2 jvi.01356-25-s0002.pdf Distribution of intrasample single nucleotide variants (iSNVs) and recombination breakpoint junctions across the POWV genome.

Fig. S3 jvi.01356-25-s0003.tif Frequency of amino acid deletion within the POW s5 RdRp.

Fig. S4 jvi.01356-25-s0004.tif Read lengths and split lengths.

Fig. S5 jvi.01356-25-s0005.tif Distribution of split read length generated by ClickSeq.

Supplemental tables jvi.01356-25-s0006.xlsx Tables S1 to S3.

ASM does not own the copyrights to Supplemental Material that may be linked to, or accessed through, an article. The authors have granted ASM a non-exclusive, world-wide license to publish the Supplemental Material files. Please contact the corresponding author directly for reuse.
