# Supplementary figures and images for: Evidence for Powassan virus deletions and defective RNA in field-collected ticks
Source: J Virol. 2026 Jan 21;100(2):e01356-25. doi: 10.1128/jvi.01356-25 (PMC12911871; doi:10.1128/jvi.01356-25)

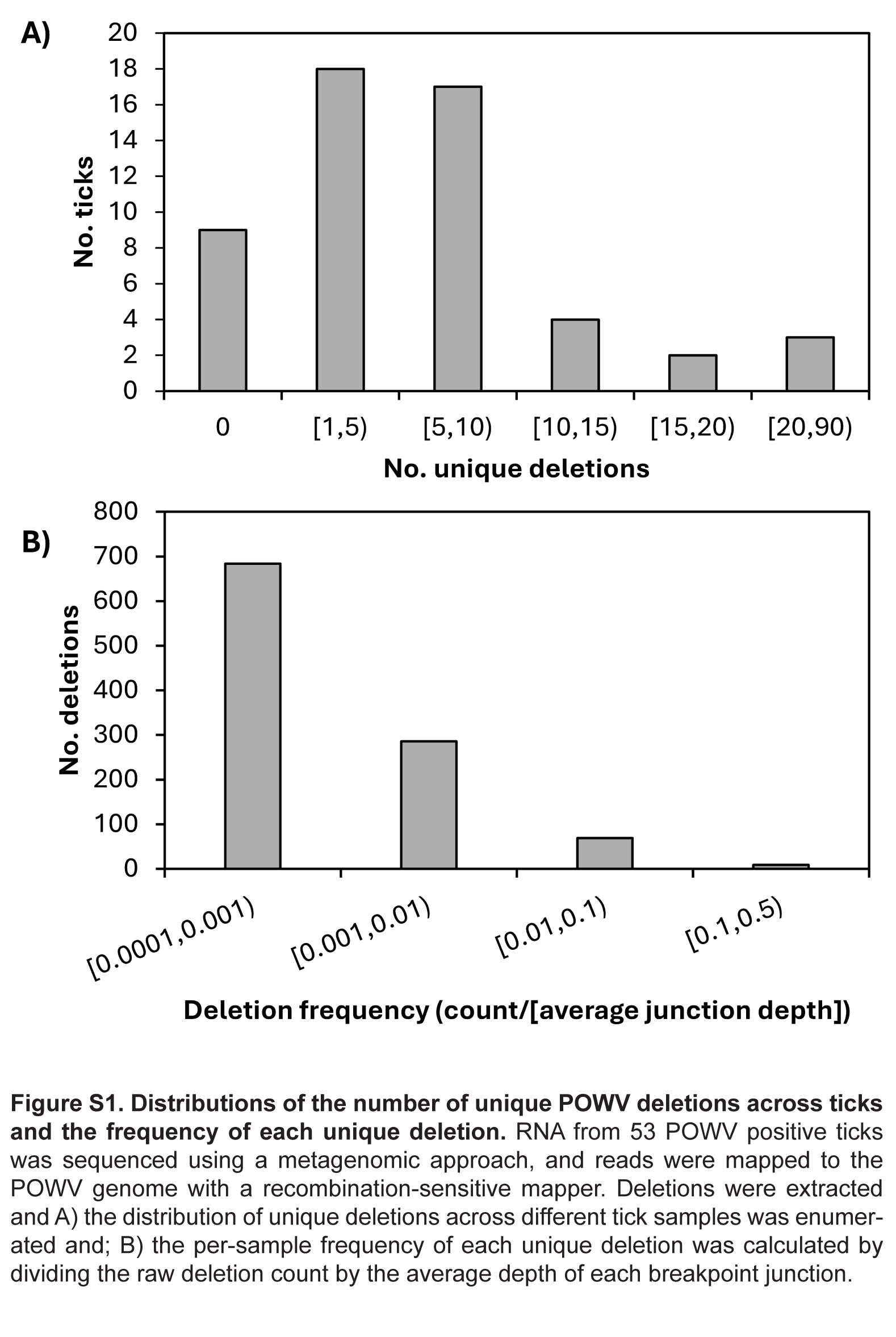

Supplement: Fig. S1 — Distributions of the number of unique POW deletions across ticks and the frequency of each unique deletion. [file jvi.01356-25-s0001.tif]

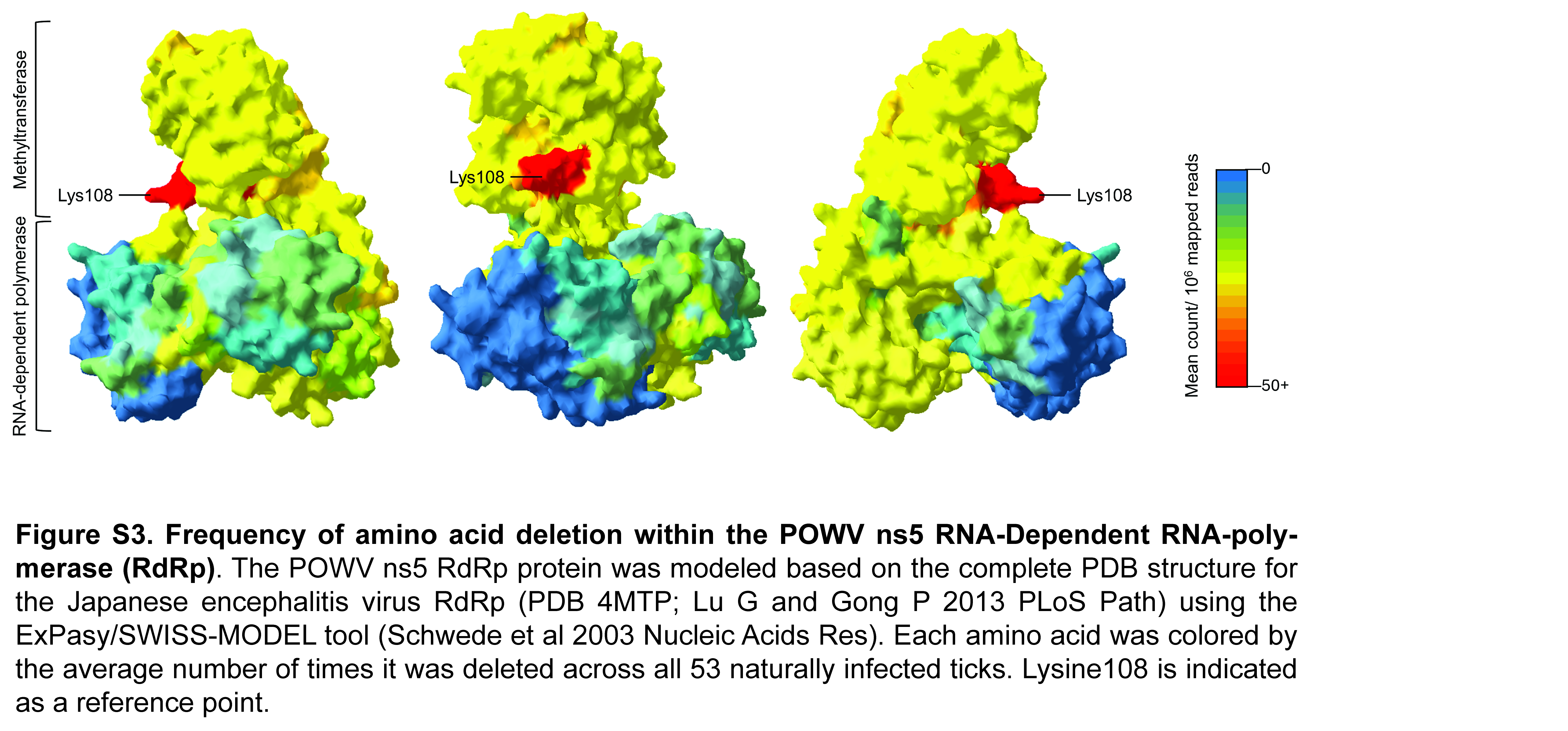

Supplement: Fig. S3 — Frequency of amino acid deletion within the POW s5 RdRp. [file jvi.01356-25-s0003.tif]

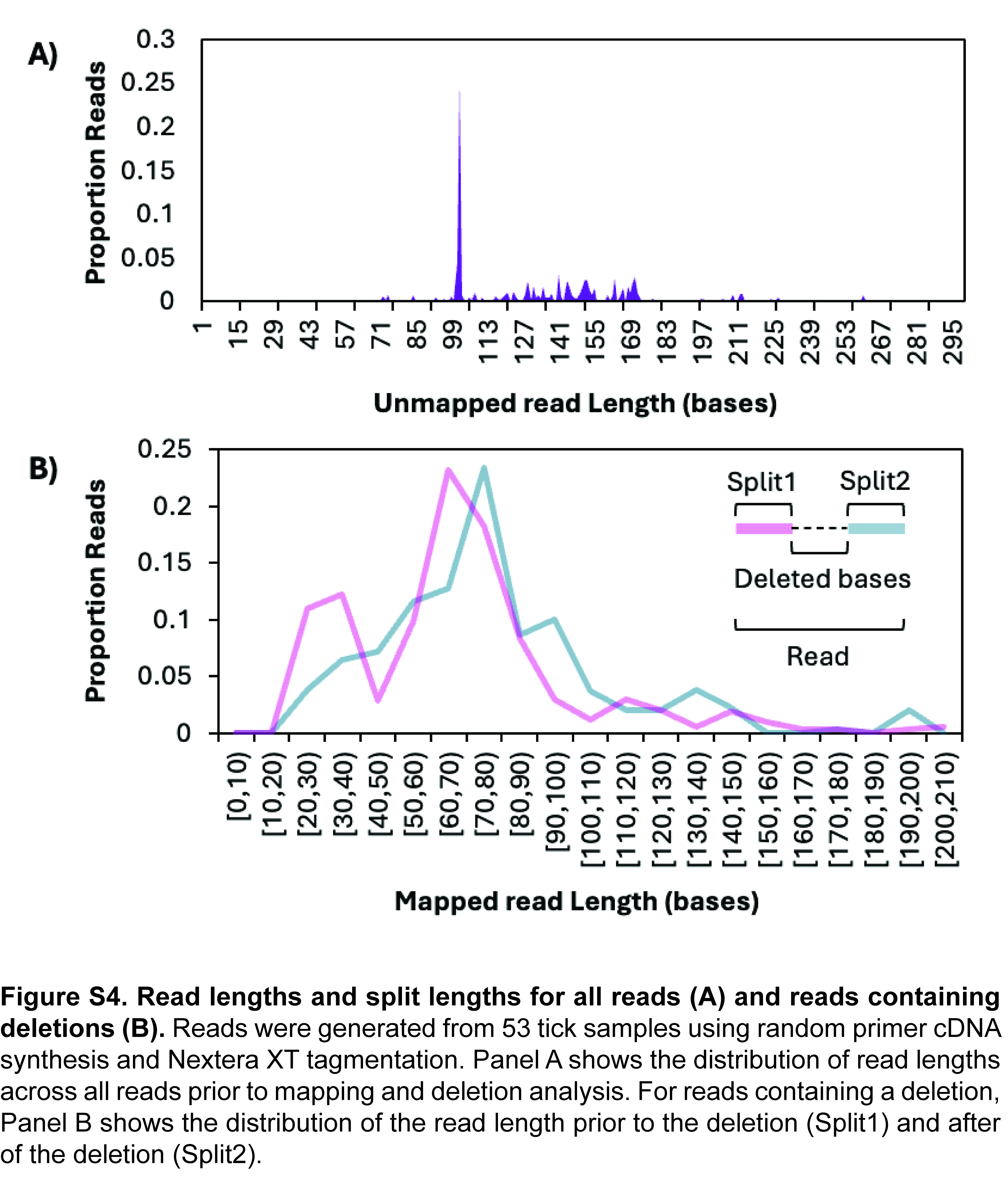

Supplement: Fig. S4 — Read lengths and split lengths. [file jvi.01356-25-s0004.tif]

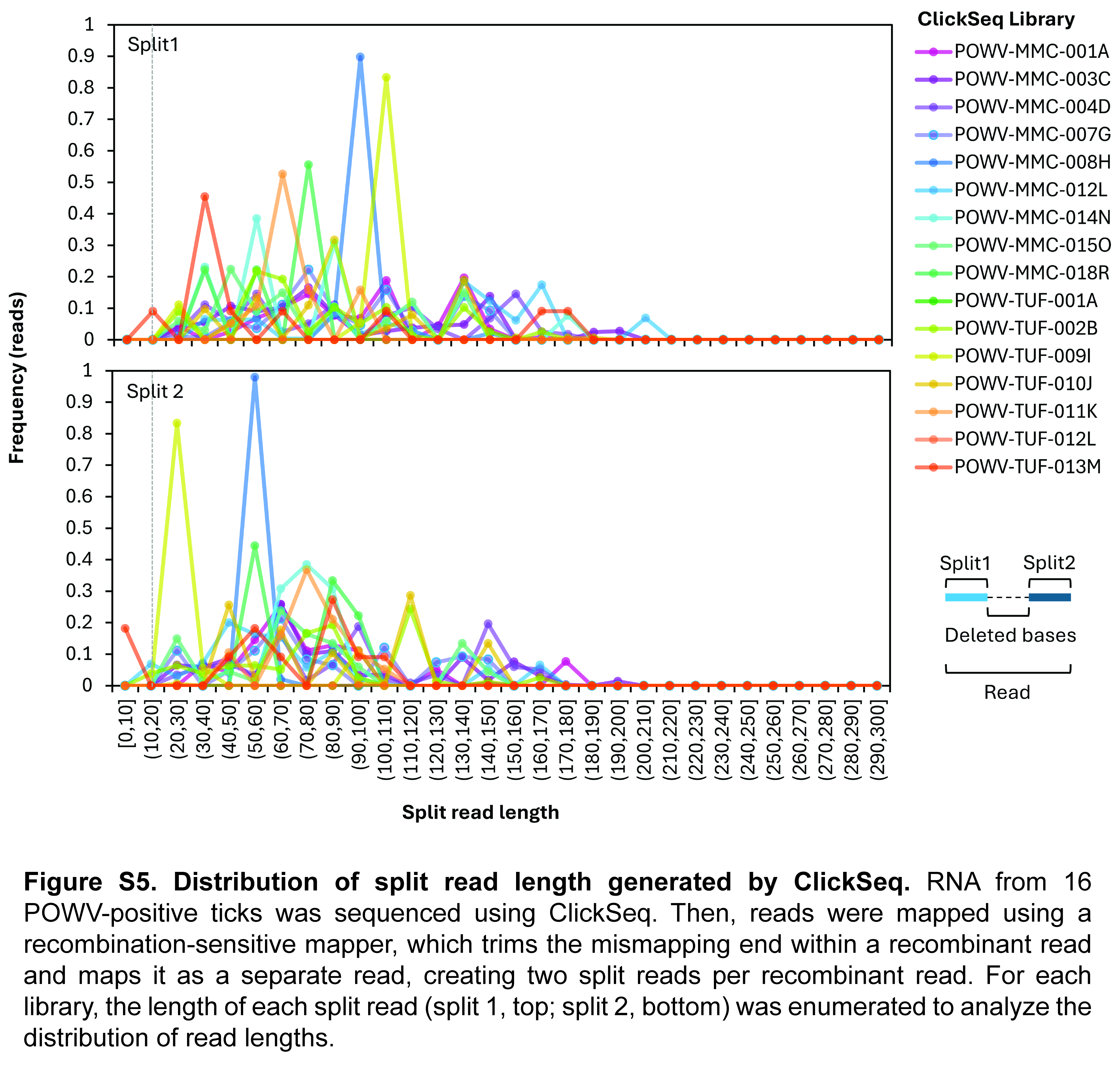

Supplement: Fig. S5 — Distribution of split read length generated by ClickSeq. [file jvi.01356-25-s0005.tif]
