# Supplementary material for: Evidence for Powassan virus deletions and defective RNA in field-collected ticks
Source: J Virol. 2026 Jan 21;100(2):e01356-25. doi: 10.1128/jvi.01356-25 (PMC12911871; doi:10.1128/jvi.01356-25)
Supplement: Fig. S2 — Distribution of intrasample single nucleotide variants (iSNVs) and recombination breakpoint junctions across the POWV genome. [file jvi.01356-25-s0002.pdf]

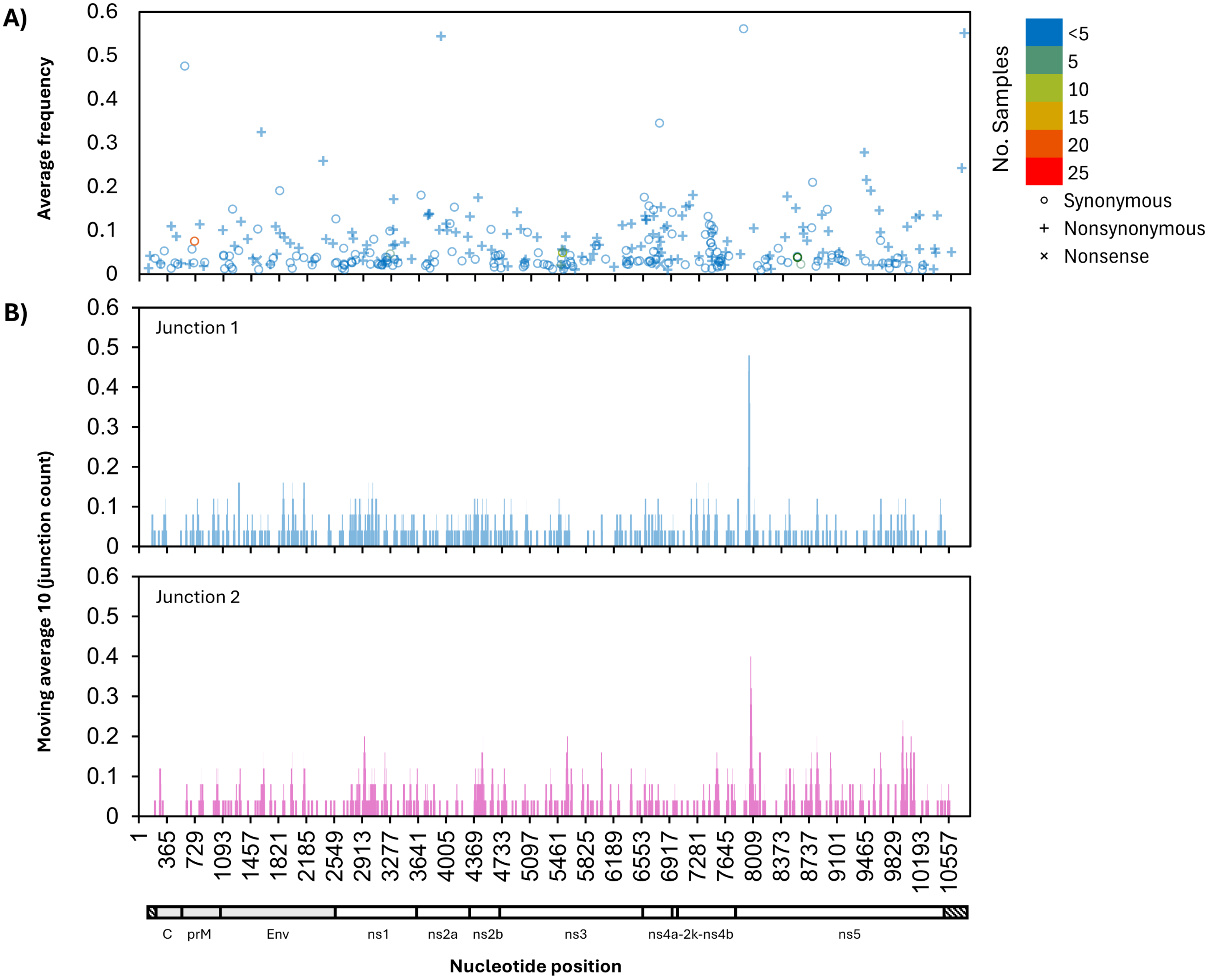

Figure S2. Distribution of intrasample single nucleotide variants (iSNVs) and recombination breakpoint junctions across the POWV genome. RNA from 53 POWV-positive ticks was sequenced using a metagenomic approach. A) Reads were mapped to the POWV genome (x-axis) using a standard mapping algorithm, and iSNVs were called using LoFreq (frequency shown on y-axis). Each symbol shows a unique iSNV, the color indicates the number of samples in which that iSNV was identified, and the shape indicates the substitution type (cross, synonymous; open circle, nonsynonymous; and x, nonsense or early-terminating). B) Reads were mapped using a recombination-sensitive mapper, deletions were extracted, the number of deletions with a 5' Junction 1 (top) and 3' Junction 2 (bottom) at each nucleotide position was enumerated, and a moving average of 10 was employed to highlight regions with increased recombination activity.
